# Urinary Metabolomic Profiling in Zucker Diabetic Fatty Rats with Type 2 Diabetes Mellitus Treated with Glimepiride, Metformin, and Their Combination

**DOI:** 10.3390/molecules21111446

**Published:** 2016-10-31

**Authors:** Yu Dong, Yi-Tao Chen, Yuan-Xiao Yang, Dan Shou, Chang-Yu Li

**Affiliations:** 1 Department of Medicine, Zhejiang Academy of Traditional Chinese Medicine, No. 132, Tianmushan Road, Hangzhou 310007, China; dongyu04428@163.com; 2 College of Pharmaceutical Science, Zhejiang Chinese Medical University, No. 548, Binwen Road, Hangzhou 310053, China; cytworld@zcmu.edu.cn (Y.-T.C.); yyx104475@163.com (Y.-X.Y.); 3 Department of Chemistry, Xixi Campus, Zhejiang University, No. 148, Tianmushan Road, Hangzhou 310028, China

**Keywords:** type 2 diabetes mellitus, Zucker diabetic fatty rats, metformin, glimepiride, combination, UHPLC/ESI-QTOF-MS

## Abstract

Type 2 diabetes mellitus (T2DM) is a high incidence metabolic disease. Glimepiride, metformin, and their combination are the most commonly used therapeutics for T2DM in the clinic, but little is known about the metabolic responses of these therapies. In this study, ultrahigh-pressure liquid chromatography/electrospray ionization quadrupole time-of-flight mass spectrometry (UHPLC/ESI-QTOF-MS)-based metabolomics was applied to detect changes in the urinary metabolomic profile of Zucker diabetic fatty (ZDF) rats in response to these treatments. Additionally, standard biochemical parameters (e.g., fasting plasma glucose, glycosylated hemoglobin, oral glucose tolerance, urinary glucose, triglyceride, total cholesterol, and insulin) and liver histopathology were monitored and observed. Six metabolites, including 3-galactosyl lactose, citric acid, sphingosine, phytosphingosine, ribothymidine, and succinoadenosine, were found significantly reverted to the normal level after these therapies. The present study is the first to present citric acid and sphinganine as the potential markers of T2DM, which could be used as indicators to observe the anti-diabetic effects of glimepiride, metformin, and their combination treatments.

## 1. Introduction

Diabetes is a global health problem that is expected to afflict 592 million people by 2035 [1], and it is associated with a large economic burden on the health systems of many countries [2]. Of this number, 90%–95% of people have type 2 diabetes mellitus (T2DM) [3]. T2DM is a metabolic disorder characterized by chronic hyperglycemia and is associated with insulin resistance, impaired insulin secretion, or both [4]. As a T2DM animal model, Zucker diabetic fatty (ZDF) rats develop hyperglycemia, hyperinsulinemia, and hypertriglyceridemia [5]. In our previous study, we successfully established a high fat and sugar diet-induced ZDF rat model. This model exhibited diabetic characteristics, and urinary metabolomic analysis of ZDF rats revealed changes in 29 endogenous metabolites, mainly associated with glyoxylate and dicarboxylate metabolism, pentose, glucuronate interconversions, and sphingolipid metabolism [6]. On the basis of this characterization, ZDF rats provide an optimal model to research the efficacy and pharmacological actions of commonly used anti-diabetic drugs such as glimepiride and metformin.

Glimepiride is a third-generation sulphonylurea that can improve both first- and second-phase insulin secretion, lower the level of plasma glucose, and inhibit the synthesis of hepatic glucose [7]. Some studies have confirmed that glimepiride can bind with the sulphonylurea receptor embedded in the pancreatic β-cell surface, which may couple with the ATP-sensitive K^+^ (KATP) channel to prompt KATP channel closure, causing Ca^2+^ influx and promoting the release of insulin [8]. Metformin is the first-line pharmaceutical treatment drug for T2DM because it specifically reduces hepatic gluconeogenesis without increasing insulin secretion, inducing weight gain, or posing a risk of hypoglycemia [9]. Initial investigations into metformin’s mechanism of action found that it is a complex I inhibitor at millimolar concentrations [10]. Much more recent studies have suggested that metformin could activate AMP-activated protein kinase (AMPK) by causing decreases in hepatic energy charge (increasing AMP:ADP or ADP:ATP concentration ratios, or both) and could suppress gluconeogenesis by inhibiting mitochondrial glycerophosphate dehydrogenase [11,12]. Usually, glimepiride is prescribed in addition to metformin to improve its anti-diabetic efficacy in clinical settings [13]. The combination of metformin and glimepiride is a well-established therapy for T2DM.

Thus far, there have been many reports about the advantages and clinical efficacy of glimepiride, metformin, and their combination treatments for T2DM patients, but the metabolic responses to these therapies have not yet been comprehensively explored. Here, we performed urinary metabolomics analysis using UHPLC/ESI-QTOF-MS to investigate the metabolic profiles and potential biomarkers of ZDF rats after these treatments. The aim of this study was to delineate the comprehensive therapeutic responses to anti-diabetic therapies and to fill gaps in the current knowledge of their precise mechanisms of action.

## 2. Discussion

DM is one of the most common chronic diseases. It is characterized by a disturbance in the intermediate metabolism of carbohydrates, proteins, and lipids and ultimately leads to chronically sustained hyperglycemia [14]. T2DM accounts for over 90% of newly diagnosed diabetes cases, and it is a major cause of heart disease, stroke and kidney failure [15,16]. Therefore, there is a desperate need for controlling this disease. To study T2DM, various animal models of diabetes, such as the ZDF rat model, have been established to improve our understanding of the efficacy and mechanisms of anti-diabetic drugs [17]. Glimepiride and metformin are the first-line of anti-diabetic drugs worldwide. Many clinical reports have indicated that the combination of metformin and glimepiride has a synergistic, anti-diabetic effect [18], but whether this synergistic effect reflects a synergism of their effects on metabolism remains completely unclear.

In the present study, we observed the obvious anti-diabetic effect of glimepiride, metformin, and their combination in ZDF rats. Treatment with their combination could reduce both body weight and urine volume to improve these typical symptoms of T2DM. Increased levels of FBG, HbA_1C_, OGTT, U-GLU, TG, and TC and the reduction in insulin were also detected in the ZDF rats induced by high fat and sugar feed. After treatment with these therapies, their combination had the best anti-diabetic effect, compared to either drug alone, and could significantly lower the levels of detected diabetic indexes, but did not influence the secretion of insulin. Meanwhile, we found that the anti-diabetic effect of metformin was better than glimepiride. Metformin could reduce the levels of FBG, HbA_1C_, OGTT, and U-GLU, but glimepiride could only decrease the level of TC in ZDF rats. Metformin as the sole anti-diabetic drug had a significant glucose lowering effect through increasing the use of glucose and inhibiting hepatic gluconeogenesis and glucose absorption [19,20]. The glucose lowering function of metformin was consistent with the results of our study in ZDF rats. However, the results also showed that glimepiride had no glucose lowering effect and did not increase the secretion of insulin, which differs from previous reports about its anti-diabetic effects [21]. The reasons underlying this difference regarding the secretion of insulin might be that long-term feeding with a high fat and high sugar diet might create excessive peroxynitrite, which damages the beta cells [22]. Some studies have also shown that the changes could be induced by the influence of beta cell mass and glucose responsiveness in the ZDF rat model [23]. Above all, the results revealed the anti-diabetic effect of glimepiride and metformin, and their combination resulted in better glucose lowering effect and lipid-lowering outcomes. The efficacies were consistent with those in clinical settings.

Secondly, we established an UHPLC/ESI-QTOF-MS-based urinary metabolomics method coupled with a multivariate statistical analysis to evaluate the metabolic changes after treatment with glimepiride, metformin, and their combination in the ZDF rat model. Urine samples were analyzed by UHPLC/ESI-QTOF-MS, operating in positive and negative ionization modes. The mass-to-charge ratio (*m*/*z*), retention time, and abundance data were subjected to PLS-DA. Our results indicated that the PLS-DA revealed a good visual separation between the control and diabetic groups, but the model group was not well separated from the glimepiride treatment group. Six distinct metabolites, including 3-galactosyl lactose, citric acid, sphingosine, phytosphingosine, ribothymidine, and succinoadenosine, exhibited changes during treatment progression. These six markers and pathway components may provide a useful, non-invasive method of clarifying the metabolic response and the anti-diabetic effect of these therapies as well as aiding in the development of more effective therapeutic approaches for T2DM through regulating metabolic pathways. Metabolic pathway analysis with MetaboAnalyst 3.0 and the KEGG, HMDB, and METLIN databases revealed three main pathological processes affected by treatment with these therapies in ZDF rats, including glyoxylate and dicarboxylate metabolism, sphingolipid metabolism, and the tricarboxylic acid cycle. On the basis of the metabolomics results, their combination therapy could regulate the most endogenous metabolites after 12 weeks, and the number and identity of the metabolites were the sum of the metabolites affected by treatment with glimepiride or metformin alone. This finding confirmed that their combination could well integrate the anti-diabetic effects of both drugs in metabolism.

Although monitoring glucose to evaluate the anti-diabetic effect is the conventional clinical method, glucose is not the only metabolite that shows altered levels in diabetes. In this study, two important metabolites, which could be used as indicators to observe the anti-diabetic effect of these treatments, were identified as citric acid and sphinganine. Citric acid plays an important role in the tricarboxylic acid (TCA) cycle and glycometabolism [24]. ZDF rats fed with high fat and sugar feed exhibited altered energy metabolism. Glimepiride and the combination of glimepiride and metformin effectively regulated the citric acid level and restored the TCA cycle. The TCA cycle is the final metabolic pathway of three nutrients and is the most important energy metabolism pathway, producing large amounts of adenosine triphosphate (ATP). Glimepiride has affinity for ATP-dependent potassium channels and can inhibit them to increase the secretion of insulin [25]. Thus, we speculated that the observed reduction in citric acid may be caused by the anti-diabetic effect of glimepiride. Sphinganine is a blocker of post-lysosomal cholesterol transport that inhibits the low-density lipoprotein-induced esterification of cholesterol [26,27]. Metformin and glimepiride can inhibit lipolysis and promote the accumulation of triacylglycerol in adipocytes. The reduction of TG and TC showed that metformin and glimepiride exhibit a marked lipid-lowering effect in ZDF rats. Sphinganine, therefore, can be used as a metabolic index component to reveal the extent of lipid lowering for different treatments of diabetes.

In summary, we evaluated the anti-diabetic effect and the urinary metabolic response after treatment with glimepiride, metformin, and their combination on T2DM in ZDF rats. On the basis of the metabolic analysis, we determined the main endogenous metabolites and concluded that the utilization of these therapies mainly influenced energy and lipid metabolism. We also identified that citric acid and sphinganine can be used as indicators to observe the anti-diabetic effect of these treatments.

## 3. Results

### 3.1. The Therapeutic Effects of Glimepiride, Metformin, and Their Combination

**Figure 1:**
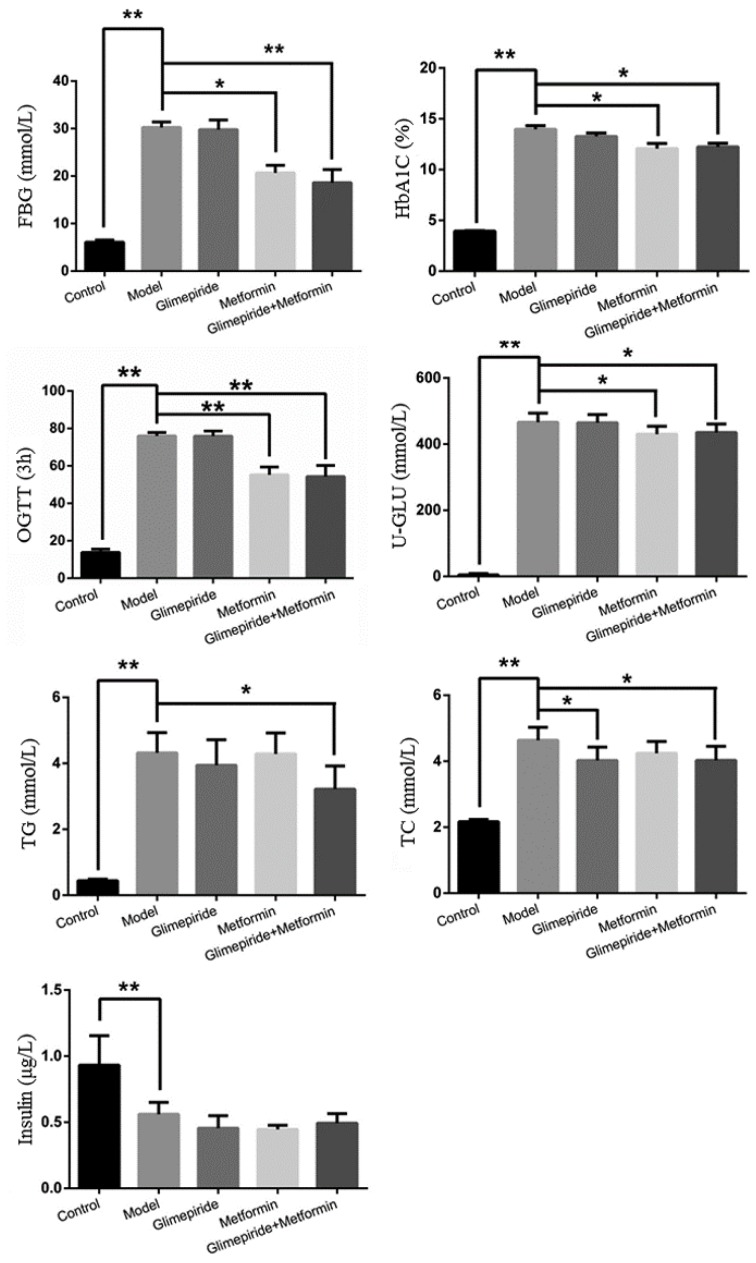
The levels of fasting plasma glucose (FBG), glycosylated hemoglobin (HbA_1c_), oral glucose tolerance (OGTT), urinary glucose (U-GLU), triglyceride (TG), total cholesterol (TC), and insulin of the control group, model group, and groups treated with glimepiride, metformin, and their combination. The values are shown as mean ± SD for six animals. * *p* < 0.05 versus values for the model group, ** *p* < 0.01 versus values for the model group.

The changes in rat body weight and urine volume after 12 weeks of treatment with these therapies are shown in Table 1. These treatments significantly reduced body weight, but only their combination decreased urine volume after 12 weeks. The levels of FBG, HbA_1c_, OGTT, U-GLU, TG, and TC were reverted with their combination treatment. The administration of metformin alone regulated the levels of FBG, HbA_1c_, OGTT, and U-GLU, while glimepiride alone only decreased the level of TC. There was no influence on the secretion of insulin after these therapies in ZDF rats. The results in Figure 1 show that metformin was more effective in reducing hyperglycemia in ZDF rats, while glimepiride was better at lowering lipid levels. Moreover, compared with the therapy with metformin or glimepiride alone, their combination treatment integrated their anti-diabetic efficacy.

### 3.2. Effects of Glimepiride, Metformin, and Their Combination Therapies on Histopathological Changes in the Liver

**Figure 2:**
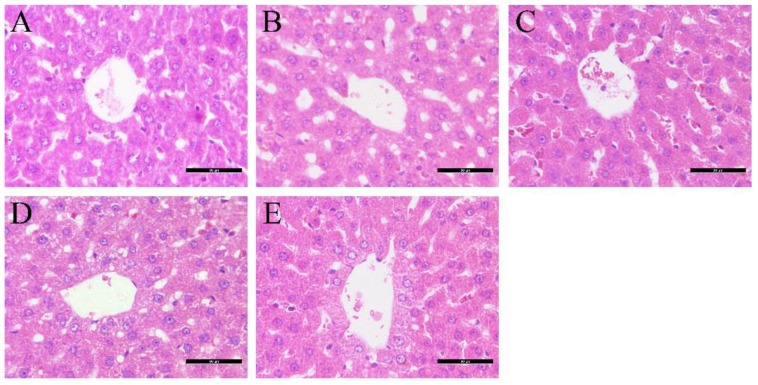
Histopathological observation of liver tissue in each group. Samples were stained with H&E and photographed at 200× magnification. (**A**) Liver of ZDF rats (fa/+); (**B**) liver of ZDF rats (fa/fa); (**C**) liver of ZDF rats (fa/fa) treatment with glimepiride; (**D**) liver of ZDF rats (fa/fa) treatment with metformin; (**E**) liver of ZDF rats (fa/fa) treatment with their combination.

As shown in Figure 2, H&E stained liver sections showed a normal liver structure of ZDF rats (fa/+) with the standard diet and injured liver structures in the rats fed with a high fat and sugar diet, indicating fat accumulation in ZDF rats (fa/fa). Histopathological analysis of the liver evaluated the approximate situation regarding the extent of liver injury after these therapies in ZDF rat models.

### 3.3. UHPLC/ESI-QTOF-MS Analysis of Urine Metabolic Profiles

**Figure 3:**
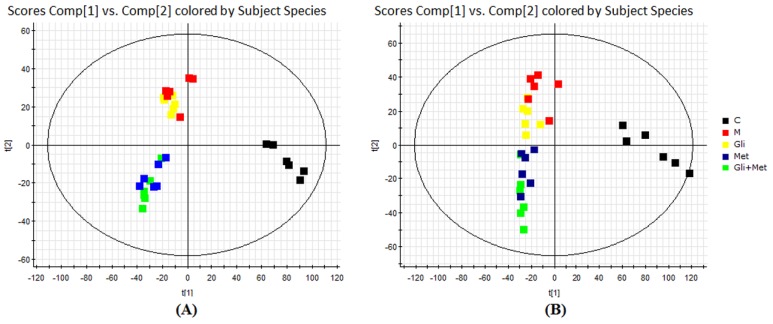
Partial least squares discriminant analysis (PLS-DA) score plots derived from the ultrahigh-pressure liquid chromatography/electrospray ionization quadrupole time-of-flight mass spectrometry (UHPLC/ESI-QTOF-MS) profiles of rat urine samples in negative (**A**) and positive (**B**) ionization modes. C: control group; M: model group; Gli: glimepiride treatment group; Met: metformin treatment group; Gli + Met: their combination treatment group.

Endogenous metabolites were well separated in the short run time of 13.0 min because of the minor particles (sub-1.8 μm) of UHPLC. All the data containing the retention time, peak intensity, and exact mass were imported to MassLynx™ software for multiple statistical analyses. Partial least squares discriminant analysis (PLS-DA) is a well-established, supervised method for multivariate statistical analysis that has been widely used in metabolomic studies. In this study, a PLS-DA method was established to distinguish the differences among the five groups and employed to identify biomarkers. The scores of PLS-DA are shown in Figure 3. The analysis showed that based on metabolite composition, the control group was obviously separated from the model and three treatment groups along latent variable 1. The differences between the three treatment groups and the model group were found along variable 2. Hence, the treatment is orthogonal to the impact of diabetes. The results also showed that after treatment with glimepiride, metformin, and their combination for twelve weeks, the ZDF rats had the trend for reverting but did not at all approach normal levels. Among the ZDF rats, glimepiride had a weaker anti-diabetic effect than metformin, and metformin played a leading role in their combination. These results were consistent with the data from the estimation of blood and urine indices.

### 3.4. Identification of Biomarkers Related to These Therapies in ZDF Rats

**Figure 4:**
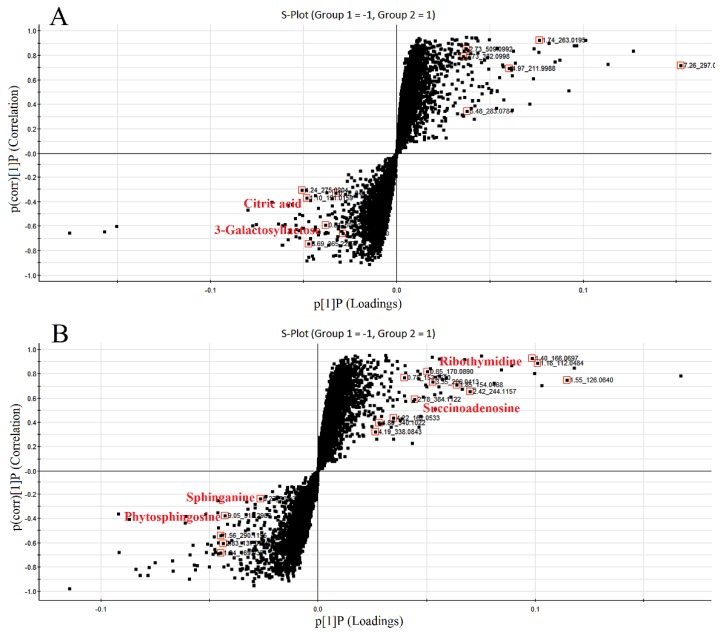
S-plots of the metabolome in rat urine from the control and model groups ((**A**), negative mode; (**B**), positive mode). The loading S-plot represents the impact of the metabolites on the clustering results. The S-plot of the OPLS-DA showed variables positively correlated with score plots. Statistically significant differences in the six metabolites responsible for the discrimination of the groups were identified between the model and treated groups. Red data points indicate the ions most responsible for the variance in the score plot.

**Figure 5:**
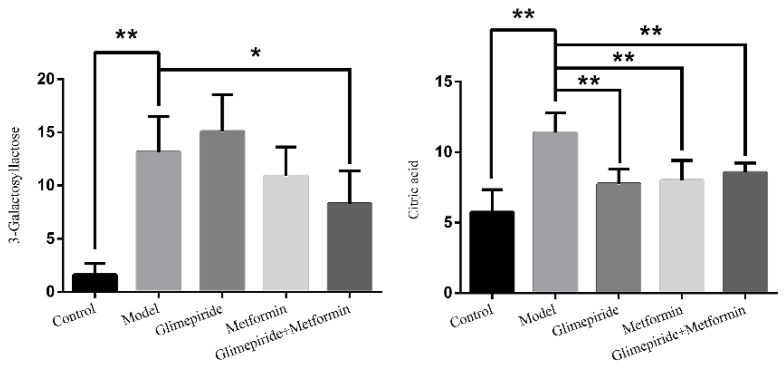

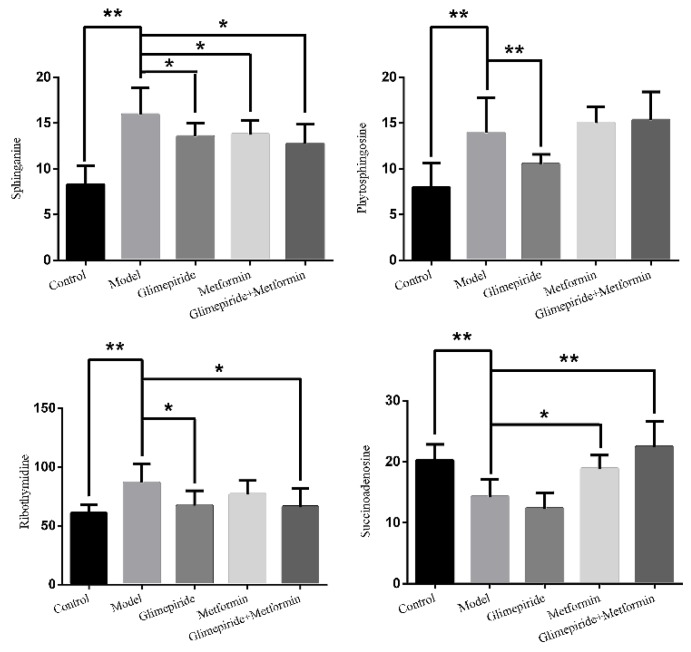
The endogenous metabolites that changed after treatment with glimepiride, metformin, and their combination. The values are shown as mean ± SD for six animals. * *p* < 0.05 versus values for the model group; ** *p* < 0.01 versus values for the model group.

The S-plots obtained from the OPLS-DA of the UHPLC/ESI-QTOF-MS data are shown in Figure 4. The distance of an ion from the origin represented its contribution to the clustering of the control and model group. A collision-induced dissociation experiment was conducted to obtain the fragmentation patterns of these potential biomarkers. The presumed molecular formulae, possible chemical constituents and potential structures of the ions were determined using the Chemspider, HMDB, and METLIN databases. A total of 29 endogenous metabolites were found significantly different between the control and model group [6]. By comparing these 29 endogenous metabolites between the model and treatment groups by the statistical method, six metabolites were found significantly reverted after these therapies. These six metabolites are shown in Table 2 and confirmed by commercial standards. The changes in the six metabolites after these therapies are shown in Figure 5.

### 3.5. Analysis of Biomarker Networks and Reconstruction of Metabolic Pathways

**Figure 6:**
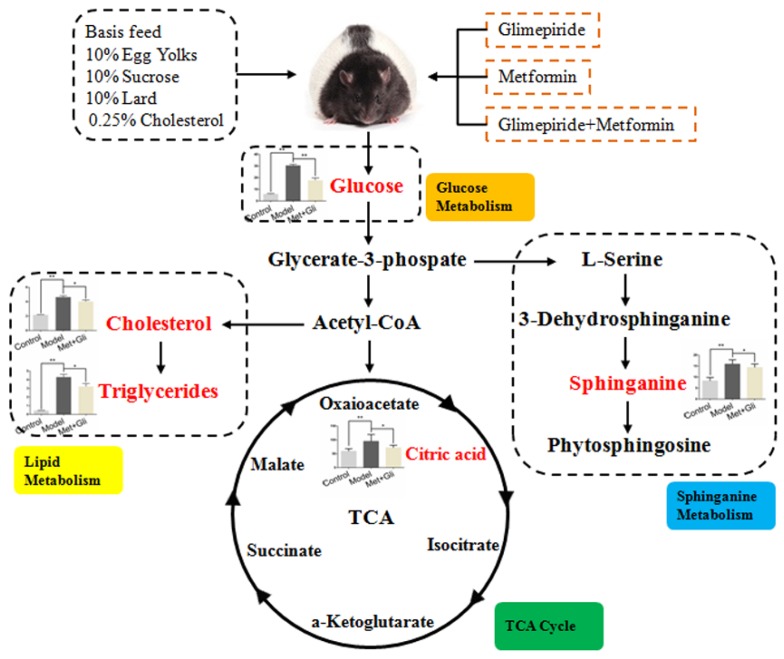
Systemic view of metabolic pathways associated with glimepiride and metformin combination treatment in obese diabetic ZDF rats (fa/fa). The network pathways were identified by the KEGG, HMDB, and METLIN databases. The red color represents the endogenous metabolites that changed when compared with the control group. Orange, yellow, green, and blue colors indicate the names of the related metabolic pathways.

The related pathways of the biomarkers were investigated by searching the KEGG and HMDB databases, and a network for the metabolites and biochemical parameters related to the anti-diabetic effects was established (Figure 6). A functional pathway analysis with MetaboAnalyst 3.0 revealed that six biomarkers were involved in three main pathological processes that were altered after these therapies.

## 4. Materials and Methods

### 4.1. Chemicals and Materials

The ZDF rats were purchased from Vital River Laboratory Animal Technology Co., Ltd. (Beijing, China). Metformin was purchased from Sino-American Shanghai Squibb Co., Ltd. (NO. 1305089, Shanghai, China). Glimepiride was purchased from Sanofi Pharmaceutical Co., Ltd. (NO. 3JB070, Beijing, China). Rat insulin ELISA Kits 10-1250-01 were obtained from Mercodia AB Co., Ltd. (NO. 21759, Uppsala, Sweden). Quo-Test A1C HbA_1c_ Reagent Kits were purchased from Quotient Diagnostics Ltd. (NO. 020166, Surrey, UK). Kits used to detect triglyceride and total cholesterol were obtained from German Desai Diagnosis System Co., Ltd. (Frankfurt, Germany). Acetonitrile and methanol (HPLC grade) were purchased from Merck (Darmstadt, Germany). Distilled water was purchased from Watson’s Food & Beverage Co., Ltd. (Guangzhou, China). Leucine encephalin was purchased from Sigma-Aldrich (St. Louis, MO, USA). Formic acid was purchased from Tianjin Kermel Chemical Reagent Co., Ltd. (Tianjin, China). All other reagents and chemicals were of analytical grade.

### 4.2. Ethics Statement

This study was carried out at the Animal Centre of Zhejiang Chinese Medicine University (SYXK 2013–0115). The study protocol was approved by the Ethics Committee of Zhejiang Chinese Medicine University. All the surgeries were performed under sodium pentobarbital anesthesia, and all efforts were made to minimize suffering.

### 4.3. Animal Handling

Six-week-old male ZDF rats (fa/fa) and ZDF rats (fa/+) were used as diabetic (model) and non-diabetic (control) groups, respectively. The room temperature was maintained at 25 °C with 60% ± 5% humidity. A 12 h light/dark cycle was used, and the rats (2 per cage) had free access to a standard diet and water. The rats were allowed to acclimatize to single housing in metabolic cages for seven weeks prior to dosing. During this period, ZDF rats (fa/+) were fed standard feed, and ZDF rats (fa/fa) were fed a high fat and sugar feed to induce diabetic symptoms. The high fat and sugar feed was composed of the standard feed with an extra 10% egg yolk, 10% sucrose, 10% lard, and 0.25% cholesterol. After acclimatization, ZDF rats (fa/+) were used as the control group. ZDF rats (fa/fa) were randomly divided into an untreated model group and three treatment groups. Each group was comprised of 6 rats. The rats in the control and model groups were administered the vehicle (0.25% sodium carboxymethyl cellulose (CMC-Na) in normal saline). The rats in the three treatment groups were administered glimepiride (5 mg/kg), metformin (200 mg/kg), and their combination (5 mg/kg glimepiride + 200 mg/kg metformin), respectively, once daily for 12 consecutive weeks.

### 4.4. Measurement of Standard Biochemical Parameters

All samples were collected 12 weeks after treatment started. The levels of fasting plasma glucose (FBG), glycosylated hemoglobin (HbA_1c_), oral glucose tolerance (OGTT), urinary glucose (U-GLU), triglycerides (TG), and total cholesterol (TC) were measured by a 7020 full automatic biochemical analyzer (Hitachi, Tokyo, Japan). Insulin level was determined using an enzyme-linked immunosorbent assay (ELISA) kit, according to the manufacturer’s instructions.

### 4.5. Observation of Liver Histopathology

The livers were fixed for 48 h in a 10% neutral formalin solution. The tissue was subsequently dehydrated with a graded ethanol series (70%–100%) and embedded in paraffin wax. The embedded tissue was sectioned (6-μm-thick sections), stained with hematoxylin and eosin (H&E), and examined by a DMI 3000B light microscope (Laica, Wetzlar, Germany) at magnifications of 200×.

### 4.6. Urinary Sample Collection and Preparation

All rats were singly housed in metabolic cages. Urinary samples were collected from the metabolic cages at ambient temperature at the 12th week after the start of treatment administration. The samples were centrifuged at 13,000 rpm at 4 °C for 5 min. The supernatants were then stored at −80 °C until analysis. All samples were thawed to room temperature and centrifuged at 13,000 rpm for 15 min before analysis. An aliquot of 2 μL was injected for UHPLC/ESI-QTOF-MS analysis after filtration through a 0.22 μm membrane filter.

### 4.7. Metabolic Profiling

Chromatography: UHPLC/ESI-QTOF-MS analysis was performed on an ACQUITY UPLC^TM^ HSS T3 column (100 mm × 2.1 mm i.d., 1.8 μm, Waters Corp., Milford, MA, USA). The optimal mobile phase consisted of a linear gradient system of (A) 0.1% formic acid in acetonitrile and (B) 0.1% formic acid in water: 0–2.5 min, 1%–11% A; 2.5–6.0 min, 11%–21% A; 6.0–8.0 min, 21%–40% A; 8.0–8.5 min, 40%–60% A; 8.5–9.5 min, 60%–99% A; 9.5–11.25 min, 99% A; 11.25–11.5 min, 99%–1% A; 11.5–13.0 min, 1% A. The column temperature was maintained at 40 °C. The flow rate of the mobile phase was 0.40 mL/min. The injection volume was 2.0 μL. In addition, a quality control (QC) sample was prepared from 10 μL of each analyzed sample. The QC sample was injected after every 6 samples.

Mass spectrometry: The optimal mass spectrometry conditions for the positive ionization mode were as follows: a capillary voltage of 2.8 kV, a desolvation temperature of 400 °C, a sample cone voltage of 40 V, an extraction cone voltage of 4.0 V, a collision energy of 6 eV, a source temperature of 110 °C, a cone gas flow of 50 L/h, and a desolvation gas flow of 550 L/h. The conditions for negative ionization mode were as follows: a capillary voltage of 2.5 kV, a desolvation temperature of 350 °C, a sample cone voltage of 35 V, an extraction cone voltage of 3.5 V, a collision energy of 6 eV, a source temperature of 110 °C, a cone gas flow of 50 L/h, and a desolvation gas flow of 500 L/h. The mass spectrometric full-scan data was acquired from 100 to 1500 Da with a 0.1 s scan time. The data was centroided, and the mass was corrected during acquisition using an external reference (LockSpray^TM^) consisting of a 0.2 ng/mL solution of leucine encephalin infused at a flow rate of 100 μL/min through a LockSpray interface.

### 4.8. Statistical Analysis

The data are expressed as mean ± SD. The differences among the levels of FBG, HbA_1c_, OGTT, U-GLU, TG, TC, and serum insulin were assessed using Welch’s *t*-tests with SPSS 19.0 (SPSS Inc., Chicago, IL, USA); values were considered different if the *p*-value was less than 0.05. The acquired mass data was analyzed with MassLynx software (version 4.1, Waters Corporation, Milford, MA, USA) for peak detection and alignment. All the data were normalized to the summed total ion intensity per chromatogram, and the resultant data matrices were processed with EZinfo 2.0 software (Waters Corp., Milford, MA, USA) using the pattern recognition approach. Metabolites were assigned by MS/MS analysis or interpreted with the following available biochemical databases: METLIN, HMDB, and KEGG. The possible biological roles of metabolites were evaluated with an enrichment analysis by MetaboAnalyst 3.0.

## 5. Conclusions

In this study, we report for the first time a comprehensive metabolic analysis after treatment with glimepiride, metformin, and their combination for T2DM in ZDF rats. UHPLC/ESI-QTOF-MS-based urinary metabolomics analysis combined with multivariate statistical analysis was applied to reveal numerous significant alterations in the levels of metabolites after these treatments. Six potential biomarkers reverted by these therapies were characterized in the negative and positive ionization modes by the MS and MS/MS information. Our findings also demonstrated that glimepiride, metformin, and their combination could reverse important diabetic indices and possibly regulate the metabolic pathways of glyoxylate and dicarboxylate metabolism, sphingolipid metabolism, and the tricarboxylic acid cycle. The anti-diabetic effects of these therapies potentially act through an ATP-dependent pathway. Citric acid and sphinganine were first considered as the potential markers of T2DM that could be used as indicators to observe the anti-diabetic effects of glimepiride, metformin, and their combination treatments.

## Figures and Tables

**Table 1:** The changes in body weight (g) and urine volume (mL) of Zucker diabetic fatty (ZDF) rats.

Index	Group	0 W	4 W	8 W	12 W
Body weight	Con	290.8 ± 14.04 *	332.6 ± 11.76 *	358.8 ± 12.95	373.6 ± 15.16
Body weight	Mod	365.0 ± 2.738	360.0 ± 9.407 *	354.8 ± 11.69	361.2 ± 12.52
Body weight	Gli	361.2 ± 4.086	337.4 ± 5.413	341.2 ± 7.190	335.0 ± 11.47 *
Body weight	Met	369.2 ± 8.288	351.4 ± 8.933	348.8 ± 9.200	329.4 ± 7.540 *
Body weight	Gli + Met	354.2 ± 21.02	345.4 ± 18.36	331.4 ± 20.12	306.0 ± 21.34 **
Urine volume	Con	11.68 ± 1.940 **	10.70 ± 3.940 **	13.56 ± 3.930 **	9.06 ± 3.380 **
Urine volume	Mod	84.68 ± 15.67	103.6 ± 15.78	108.3 ± 10.13	96.68 ± 12.19
Urine volume	Gli	79.12 ± 10.29	69.68 ± 18.20 **	97.38 ± 15.95	80.44 ± 23.04
Urine volume	Met	72.68 ± 19.39	77.58 ± 16.94 *	71.60 ± 14.63 *	82.96 ± 1.100
Urine volume	Gli + Met	83.54 ± 7.450	79.06 ± 16.84 *	72.10 ± 11.97 **	65.94 ± 10.81 *

* Compared with the model group, *p* < 0.05; ** compared with the model group, *p* < 0.01.

**Table 2:** Identified biomarkers in ZDF rat urine after treatment with glimepiride, metformin, and their combination compared with the model group.

Time	Mass	ppm	Formula	Mass Fragments	Structure	Compound
0.81	503.1589	−4.6	C_18_H_32_O_16_	509 [M − H]^−^, 383 [M − H − C_4_H_8_O_4_]^−^, 323 [M − H − C_6_H_12_O_6_]^−^, 221 [M − H − C_10_H_18_O_9_]^−^, 110 [M − H − C_12_H_24_O_14_]^−^, 89 M − H − C_15_H_26_O_13_]^−^	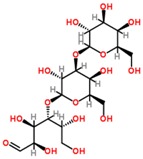	3-Galactosyl lactose
1.10	191.02	4.2	C_6_H_8_O_7_	191 [M − H]^−^, 173 [M − H − OH]^−^, 129 [M − H − CH_2_O_3_]^−^, 111 [M − H − CH_4_O_4_]^−^, 85 [M − H − CH_6_O_4_]^−^, 67 [M − H − C_2_H_4_O_6_]^−^	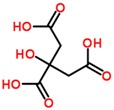	Citric acid
2.75	382.0994	−1.3	C_14_H_17_N_5_O_8_	382 [M − H]^−^, 364 [M − H − H_2_O]^−^, 302 [M − H − CH_4_O_4_]^−^, 266 [M − H − C_4_H_4_O_4_]^−^, 206 [M − H − C_6_H_8_O_6_]^−^, 133 [M − H − C_9_H_7_N_5_O_4_]^−^	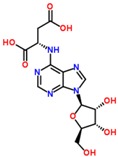	Succinoadenosine
0.81	259.092	−3.9	C_10_H_14_N_2_O_6_	259 [M + H]^+^, 242 [M + H − NH_3_]^+^, 215 [M + H − CH_2_NO]^+^, 127 [M + H − C_5_H_10_NO_3_]^+^, 81 [M + H − C_5_H_10_N_2_O_5_]^+^	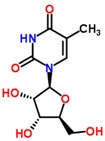	Ribothymidine
9.05	318.3012	1.3	C_18_H_39_NO_3_	318 [M + H]^+^, 300 [M + H − H_2_O]^+^, 261 [M + H − C_4_H_9_]^+^, 256 [M + H − C_2_H_8_NO]^+^, 228 [M + H − C_5_H_14_O]^+^, 102 [M + H − C_13_H_30_NO]^+^	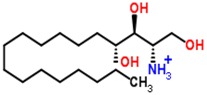	Phytosphingosine
9.27	302.3071	4.0	C_18_H_39_NO_2_	302 [M + H]^+^, 284 [M + H − H_2_O]^+^, 258 [M + H − C_2_H_4_O_3_]^+^, 240 [M + H − C_2_H_6_O_4_]^+^	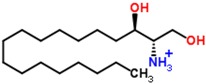	Sphinganine
